# Incidence and predictors of neonatal mortality among neonates admitted in Amhara regional state referral hospitals, Ethiopia: prospective follow up study

**DOI:** 10.1186/s12887-020-02031-x

**Published:** 2020-04-01

**Authors:** Banchigizie Adane Mengistu, Ayenew Engida Yismaw, Zelalem Nigussie Azene, Muhabaw Shumye Mihret

**Affiliations:** 1 Teda Health Science College, Amhara Regional Health Bureau, Department of Midwifery, Po. Box 196, Gondar, Ethiopia; 2 Department of Clinical Midwifery, School of Midwifery, College of Medicine and Health Sciences, University of Gondar, Gondar, Ethiopia; 3 Department of Women’s and Family Health, School of Midwifery, College of Medicine and Health Sciences, University of Gondar, Gondar, Ethiopia

**Keywords:** Amhara region, Neonatal intensive care unit, Neonatal mortality incidence rate, Neonatal death, Predictors, Survival of neonates

## Abstract

**Background:**

Globally, about 2.7 million neonates die annually and more than 99% of these deaths happened in developing countries. Although most neonatal deaths are preventable and attempts had been taken to tackle these deaths, an aggregate of 30 neonatal deaths per 1000 live births had been reported in Ethiopia. In this regard, identifying the predictors could be an important step. However, evidence on the incidence and predictors of neonatal mortality has been limited in Ethiopia, in the study area in particular. Even the available studies were limited in scope and were retrospective or cross section in nature. Thus, this study is aimed at assessing the incidence and predictors of neonatal mortality among neonates admitted in Amhara regional state referral hospitals, Ethiopia..

**Method:**

A multi center prospective follow up study was conducted on 612 neonates admitted in Amhara region referral hospitals from July 01 to August 30, 2018. A simple random sampling technique was used to select three of all referral hospitals in the study settings and all neonates admitted in the selected hospitals were included. Data were entered into Epi info version 7.0 and exported to STATA 14.0 for analysis. Cox proportional hazard regression model was fitted to identify predictors of neonatal mortality. Crude and Adjusted hazard ratio with 95% confidence interval was computed and variables’ statistical significance was declared based on its AHR with 95% CI and *p*-value ≤0.05.

**Result:**

Overall, 144 (18.6%) neonates died with a total person-time of 4177.803 neonate-days which is equivalent to the neonatal mortality rate of 186 per 1000 admitted neonates with 95% CI (157,219). The incidence rate of neonatal mortality was 27 per 1000 admitted neonates with 95%CI (23, 33). Maternal age ≥ 35 years (AHR = 2.60; 95%CI: 1.44, 4.72), mothers unable to read and write (AHR = 1.40; 95%CI: 1.23, 2.44), multiple pregnancy (AHR = 3.96; 95%CI: 2.10, 7.43) and positive maternal HIV status (AHR = 6.57; 95%CI: 2.53, 17.06) were predictors of neonatal mortality.

**Conclusion:**

In this study, the neonatal mortality rate was higher than the national figure. Its most predictors were found to be modifiable. Thus, the stakeholders would better consider the aforementioned predictors to decrease this higher burden.

## Background

Neonatal Mortality Incidence Rate (NMIR) is a probability of dying during the first 28 days of life and expressed per 1000 admitted neonates [1, 2]. The neonatal period commences at birth and ends at 28 completed days after birth and this period is the most vulnerable time for a child’s survival [3–5]. This period is also further classified as early (the first 7 days) and late (the remaining 21 days) neonatal period and majority neonatal mortality occur in the early neonatal period [4, 6, 7].

Neonatal mortality (NM) is a major global public health challenge [5, 8, 9]. Approximately 2.7 million neonates worldwide each year die in the first month of life and most of these deaths happen in developing countries [6, 10]. NM accounts for about 44% of under-five deaths and more than 99% of these deaths occur in middle or low-income countries [5, 6]. According to United Nations International Children’s Emergency Fund (UNICEF) 2018 report, Neonatal Mortality Rate (NMR) in the globe and the least developed countries was 19 and 26 per 1000 live birth respectively [1]. Accordingly, the problem is much higher in South Asia (SA), West and Central Africa (WCA), and sub-Saharan Africa (SSA) [1]. Similarly, NMR in Ethiopia is higher (i.e., reported to be 30 per 1000 live births) and the largest figure was recorded at Amhara national regional state where it was estimated to be about 47 per 1000 live birth [2, 11].

To alleviate these problems, efforts have been made globally as well as nationally. For instance, it was included in and remained ‘unfinished agenda” of Millennium Development Goals (MDGs) which was extended to Sustainable Development Goals (SDGs) [9, 12]. Hence, target 2 of SDG 3 is planned to end preventable deaths of newborns by all countries aiming to reduce neonatal mortality to at least as low as 12 per 1000 live births by 2030 [9]. In line with this, Ethiopia has planned to reduce NMR from 28 in 2015/16 to 10 per 1000 live births by 2019/2020 [13]. Consequently, the NMR has went down from 36 deaths per 1000 live births in 1990 to 19 in 2015, and the number of neonatal deaths declined from 5.1 million to 2.7 million globally [10]. Generally, the number of children dying before the age of five has declined from 9.9 million in 2000 to 5.6 million in 2016 [3]. However, the proportion of under-five deaths in the newborn period has increased from 41 to 46% between 2000 and 2016 globally [3]. Moreover, the decline in neonatal mortality (47%) from 1990 to 2015 has been slower than that of post-neonatal under-five mortality (58%) globally [3]. The scenario is similar in Ethiopia, where NMR has decreased from 49 in 2000 to 29 in 2016 as to EDHS reports [2]. However, the NMR decrement in Ethiopia is also much slower than that of post neonatal under-five mortality (41% compared with 85%) from 2000 to2016 [2]. Thus, identifying the causes of such challenges is one import part of the efforts. The previously identified causes of neonatal mortality such as hypothermia, neonatal sepsis, respiratory distress syndrome, and prenatal asphyxia may vary across settings and over time [12, 14–19].

The high NMR reflects the poor quality of care service especially during the perinatal period in a given country or region [3]. Hence, its implication reminds the concerned bodies to evaluate their respective health care system and to develop effective strategies. To design such evidence-based and problem oriented strategies which may be effective - for instance, in reducing delays in accessing obstetric and neonatal health care services such as improving community awareness of neonatal death risk factors, cultivating practices towards birth preparedness and complication readiness plan, reducing transportation barriers, preventing financial barriers, strengthening referral mechanisms, reducing barriers to good health-seeking behaviors, and enhancing the level of health facility’s preparedness and readiness for neonatal obstetric emergencies, the role of accurate contemporize information is compulsory for combating the burden thereby for achieving the SDG. Thus, methodologically sound and representative investigations need to be done by far, more importantly in the settings where high NMR has been reported, and the Amhara national regional state is an example. Having this insight, this study was conducted to assess incidence and predictors of neonatal mortalities among neonates admitted in Amhara region referral hospitals, Northern Ethiopia, 2018.

## Methods

A multicenter prospective follow up study was conducted in referral hospitals which are located in Amhara national regional state, Northern Ethiopia from July 01 to August 30/2018.

Amhara national regional state is the second-largest and populous region in Ethiopia [2]. The region has five referral hospitals namely University of Gondar comprehensive specialized hospital (UoGCSH), Felege-Hiwot comprehensive specialized hospital (FHCSH), Dessie referral hospital (DRH), Debre-Markos referral hospital (DMRH) and Debre-Birhan referral hospital (DBRH). Among these, Dessie, Felegehiwot and Debre-Markos referral hospitals were selected randomly.

Thereafter, we included all neonates who had been admitted in the corresponding neonatal intensive care unit (NICU) of each selected hospital.

We calculated the sample size for this study by using STATA version14 with the following assumptions: the probability of neonatal death-0.88 and the hazard ratio of the predictor breast feeding-7.5 as reported in a previous study done in Tigray, Northern Ethiopia [12]; power-80%; confidence level-95%; margin of error-4% and non-response rate-10%. Accordingly, the final sample size was obtained to be 631.

The number of sample size for each selected hospital was allocated proportionally based on the number of previous cases flow. All recruited study participants had been followed till the outcome of interest (i.e. either death or censure) appeared. Data were collected through face to face interview and chart review. The outcome variable of the study was admission outcome which was dichotomized as death (coded as ‘1’) or censored (coded as ‘0’) whereas, the explanatory variables included socio-demographic, obstetrics, medical, health service and neonatal related factors. These include maternal age, mother’s educational status, husband’s educational status, marital status, religion, maternal occupation, husband’s occupation, ethnicity, residence, distance from nearest health facility, premature rupture of membrane (PROM), delivery complication, mode of delivery, parity, Infant of Diabetic Mother (IDM), history of previous dead fetus, polygamous household, number of ANC visit, HIV status of mother, tetanus toxoid vaccine, multiple pregnancies, birth interval, skilled birth attendant, place of ANC visit, place of delivery, birth weight, congenital abnormality, fetal presentation, 1st minute APGAR score, 5th minute APGAR score, time of initiation of breastfeeding, hypothermia, neonatal sepsis, respiratory distress syndrome (RDS), jaundice, perinatal asphyxia (PNA), anemia, hypoglycemia, prematurity, neonatal sex, age on admission, neonatal complication, feeding option, cry at birth and weight at admission.

### Operational definitions

#### Time

the period from starting of observation until the occurrence of outcome of observation (death or censored).

#### Death

In this study, death referred to the study subject who had experienced the interest of event (had died) during the observation period and labeled as (1).

#### Censored

In this paper, censored is referred to the study subject who had not experienced death during the follow-up period. These included neonates who had lost follow up, referred to other health institution, discharged with improvement or stayed with admission beyond 28 days of neonatal age.

### Data collection, quality control mechanism, processing, and analysis

Data were collected through face to face interview and chart review using structured questionnaire and checklists which were adapted from related literature [12, 17]. The questionnaire was prepared in English and translated into Amharic and then re-translated back to English.

About six health personnel had been recruited for the data collection process. These included three professional diploma midwives for data collection and another three professional first degree midwives for supervision. A training of 2 days had been provided for the data collectors and supervisors regarding the ethical issues, general approaches, strategies to minimize information bias, etc. Pretest then had been conducted on the 5% of the total sample sizes in the unselected referral hospital prior to the commencement of actual data collection. During the data collection period, all neonates admitted in NICU with their mothers were enrolled in the first month and then had been followed until the occurrence of the outcome of interest. After putting aside the baseline data on socio-demographic factors; obstetrics related factors; health service-related factors and neonatal factors on the first day of enrollment, gathering data on neonatal related factors had been carried out daily throughout the follow-up period (i.e. from the time of admission to the 28 days of neonatal age) or till the neonates had either experienced the interest of event (i.e. death or censored). In the mean time, data collectors had been supervised, samples of respondents re-interviewed and the results then cross-checked.

Moreover, each data collector had checked the questionnaires and the checklists for completeness before leaving each study participant. Each questionnaire and checklist was then reviewed daily by supervisors and the principal investigator to check for completeness and clarity. Data entry format template, using Epi info version 7, was produced and data were entered.

Data clean up was done before analysis. Data were checked, coded and entered to EPI INFO version 7 then it was exported to STATA version 14 for analysis. Both descriptive and analytical statistical procedures were utilized. Descriptive statistics like percentage, median, interquartile range (IQR), mean and standard deviation were employed. Tables and fingers were also used for data presentation.

**Fig. 1:**
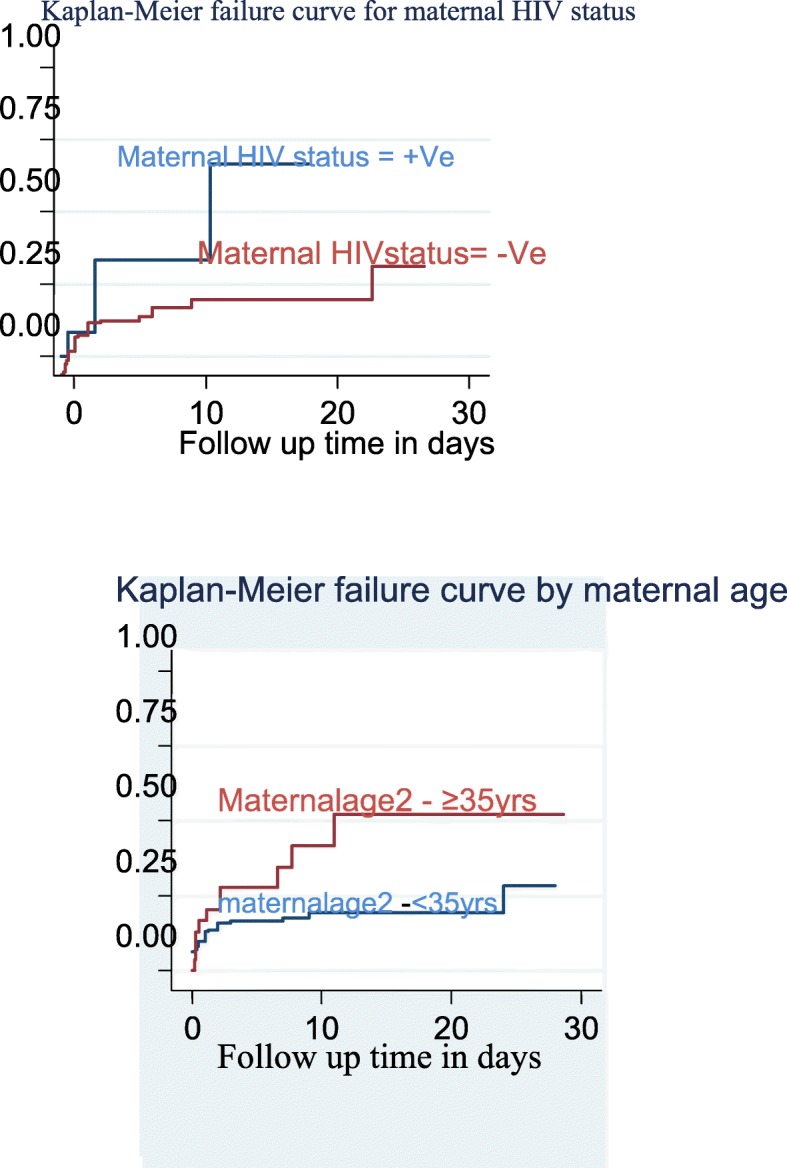
Kaplan-Meier failure curve for neonates admitted in NICU by maternal HIV status and maternal age respectively at Amhara referral

**Fig. 2:**
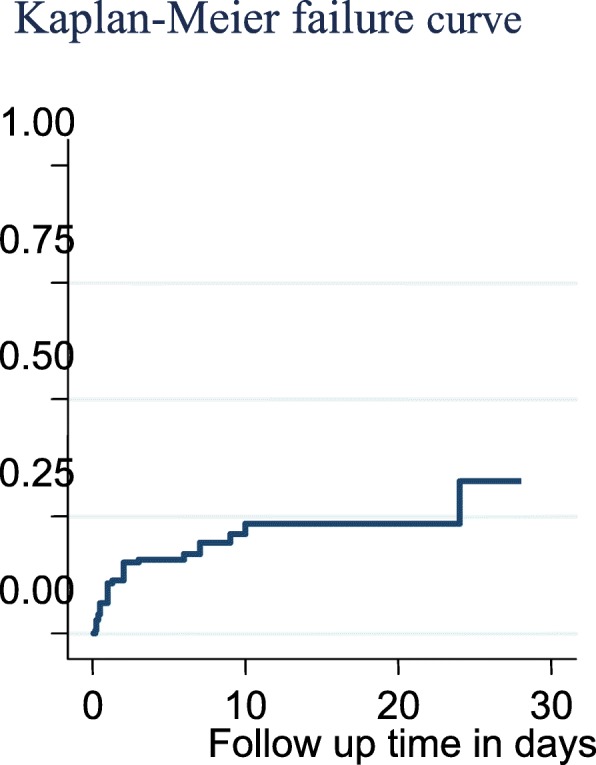
Kaplan-Meier failure curve for neonates admitted in NICU at Amhara region referral hospital. The graph shows the proportion of neonatal death during follow up time

**Fig. 3:**
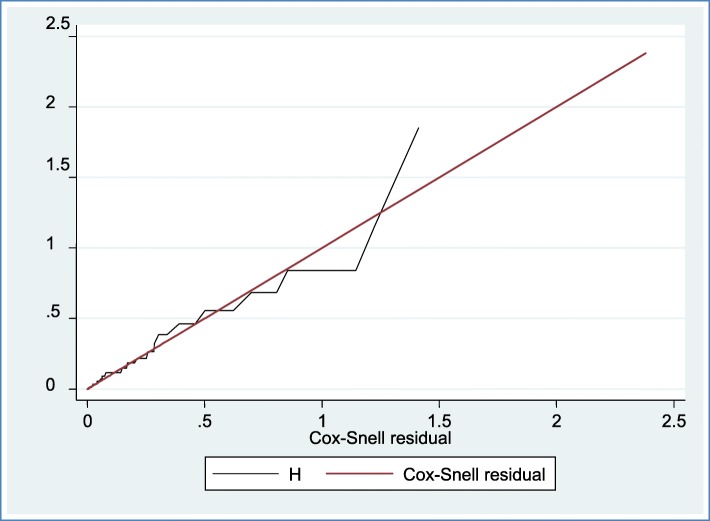
Cox-Snell residual Nelson- Alen cumulative hazard graph on neonates admitted in NICU at Amhara referral hospital, Northwest Ethiopia, 2018 for the goodness of model fitness which shows the hazard function follows the 45^o^ closed to the baseline

Proportional hazard assumption was checked both graphically **(**Fig. 1**))** and hypothetically using a hypothesis test called Shoenfield residual test (global test). Accordingly, Shoenfield residuals test showed that the proportional hazard assumption was satisfied. After the proportional hazard assumption had been checked, both bivariate and multivariable Cox proportional hazard regression analysis was computed. All variables having *P*-value ≤0.2 and with no missing values in the bivariable analysis had been then further fitted to the final model to identify independent predictors of neonatal mortality and finally the variables which have independent association with outcome variable were identified on the basis of AHR, with 95% CI and *p*-value ≤0.05. In addition, Kaplan Meier Estimator curve was used to estimate failure time of neonate (Fig. 2), and Cox-Snell residual test was utilized to check the goodness of model fitness (Fig. 3).

## Result

### Socio-demographic and economic characteristics

A total of 612 mother-neonate pairs were included in the study making a response rate of 97%. Majorities (78.1%) of the mothers of neonates were in the age group of 20–34 years with a median of 27 years with IQR = 24–31. More than half (62.58%) of the respondents were urban dwellers and nearly nine-tenths (89.22%) of the respondents were Orthodox Christian by religion. Almost all (96.9%) of the mothers were married and about 594 (97.06%) of respondents belong to Amahara Ethnicity. Nearly four-fifth (77.1%) of respondents’ family earned less than $139 monthly. About one-third (33.82%) of mothers had not been able to read and write. More than half (58.17%) of the mothers and more than one-third (38.45%) of the husbands were housewives and farmers by occupation respectively (Table 1).

**Table 1:** Socio-demographic and economic characteristics of parents of neonates admitted in Amhara Referral hospitals Northern Ethiopia (*n* = 612)

Variables	Number	Percent
Current Maternal Age		
18–19 years	26	4.25
20–34 years	478	78.10
≥ 35 years	108	17.65
Marital status		
married	593	96.9
unmarried	19	3.1
Maternal Occupation		
Housewife	356	58.17
Government employee	141	23.04
Merchant	77	12.5
Others^a^	38	6.21
Husband Occupation (*n* = 593)		
Farmer	228	38.45
Government employee	159	26.81
Merchant	133	22.43
Others^b^	73	12.31
Maternal educational status		
Unable to read & write	207	33.82
Able to read & write	30	4.9
Primary school(1–8)	107	17.48
Secondary school (9–12)	76	12.42
Higher education (>12)	192	31.37
Husband educational status (*n* = 593)		
Unable to read & write	177	29.85
Able to read & write	65	10.96
Primary school	63	10.62
Secondary school	89	15.01
Higher education	199	33.56
Family monthly income (in US$)		
< 139	472	77.1
≥ 139	140	22.9
Religious		
Orthodox	546	89.22
Muslim	66	10.78
Distance to health facility in hours (on foot)		
≤ 2 h	586	95.8
> 2 h	26	4.2
Ethnicity Group		
Amhara	594	97.06
Others^c^	18	2.94

^a^Student, Daily laborer, House worker & Private employee, ^b^Daily laborer, Student, Private employee & Car driver, ^c^Agew, Oromo & Binshangul Gumz

### Obstetrics and medical-related characteristics of the respondent

More than two-fifth (42.97%) and nearly one-fourth (12.9%) of mothers were primiparous and grand-multiparous respectively. Regarding birth spacing between the previous and the indexed pregnancy, majority (83.7%) of respondents had an inter-pregnancy interval of ≥24 months. More than a quarter (29.24%) of the respondents gave birth through cesarean section. All (100%) parents of neonates reported to have monogamous sexual relationship. Substantial member (93.79%) of the mothers of neonates had ANC follow up, whereas significant proportion (53.66%) of the mothers had incomplete ANC visit in the indexed pregnancy. Almost one-fifth (19.1%) of the mothers of neonates had a history of PROM, whereas more than one-tenth (10.5%) of the mothers conceived multiple fetuses in the indexed pregnancy. About 18 (2.94%) and 108 (17.65%) of the mothers of the neonates had Diabetes Mellitus in the indexed pregnancy and history of a previous dead fetus respectively (Table 2).

**Table 2:** Obstetric and medical-related factors among mothers of neonates admitted in Amhara region referral Hospitals, Northern Ethiopia, 2018 (*n* = 612)

Variables	Number	Percent
Parity		
1	263	42.97
2–4	270	44.12
≥ 5	79	12.91
Interpregnancy interval in months (*n* = 332)		
< 24	51	15.4
24–120	278	83.7
> 120	3	0.9
Delivery complication in the indexed pregnancy		
Yes	323	52.78
No	289	47.22
Mode of delivery		
Cesarean section	179	29.25
Spontaneous vaginal delivery	357	58.33
Forceps delivery	11	1.80
Vacuum delivery	34	5.56
Vaginal with episiotomy	27	4.41
Assisted breech delivery	4	0.65
Maternal HIV status		
Positive	15	2.5
Negative	597	97.5
Tetanus toxoid vaccine for the index pregnancy		
Yes	504	82.4
No	108	17.6
Number of ANC visit (*n* = 574)		
One visit	16	2.79
Two visit	109	18.99
Three visit	183	31.88
Four and more visit	266	46.34

### Health service-related characteristics

Out of 397 birth attendants with identified professions, about 171 (43.07%) of them were midwives. Among 591 health facility deliveries, nearly two-third (68.19%) of them were undertaken at hospitals. More than half (60.29%) of the mothers of neonates visited health centers for ANC follow up during the indexed pregnancy **(**Table 3**).**

**Table 3:** Health service related factors of respondents in Amhara region referral hospitals, Northern Ethiopia, 2018 (*n* = 612)

Variables	Numbers	Percent (%)
Birth attendants (*n* = 397)		
Diploma midwifery	34	8.56
BSc midwifery	110	27.71
MSc midwifery	27	6.80
IEOS	16	4.03
Intern	32	8.06
MD/GP and above	154	38.79
Others^a^	24	6.05
Place of delivery		
Home	21	3.43
Health facility	591	96.57
Level of health facility for place of delivery (*n* = 591)		
Health post	3	0.51
Health center	151	25.55
Primary hospital	93	15.74
Referral hospital	310	52.45
Private clinic	34	5.75
Place of ANC visit (*n* = 574)		
Health post	28	4.58
Health center	369	60.29
Hospital	122	19.93
Private	55	8.99

*IEOS* Integrated Emergency in Obstetrics and Surgery, *MD* Medical Doctor, *GP* General Practitioner

^a^Health extension workers & Traditional birth attendants

### Fetal and neonatal related characteristics

The substantial proportion (59.6%) of neonates were males by sex and more than half (62.07%) of the neonatal weight ranged between 2.5 kg and 4 kg with mean ± SD weight of 2.56 (±0.766). The majority (91.50%) of the neonates were delivered with the normal presentation. The first and fifth minute Apgar score of the neonates were found in the range of 2 to 9 with a mean ± SD of 6.31(±1.737) and 4 to 10 with mean of 7.63 (±1.437) respectively. About 568 (92.81%) of the mothers of the neonates had a plan for exclusive breastfeeding, whereas about 40 (6.54%) of the respondents intended to practise mixed infant feeding. Among mothers with exclusive breast feeding plan, nearly half (48.68%) of t them had initiated neonatal feeding within 1 h of birth. More than two-third (69.3%) of the neonates had been admitted in NICU within 24 h of birth. About 186 (30.4%) of the neonates had experienced complication at the time of birth and nearly one - third (60.3%) of neonates cried at birth (Table 4).

**Table 4:** Fetal and neonatal characteristics of the neonates admitted in Amhara region referral hospitals, Northern Ethiopia, 2018 (*n* = 612)

Variables	Number	Percent
Birth weight known		
Yes	580	94.77
No	32	5.23
Birth weight in kg (*n* = 580)		
< 2	144	24.83
2–2.5	76	13.10
2.5–4	360	62.07
Fetal presentation		
Cephalic	560	91.50
Breech	48	7.84
Shoulder	4	0.65
APGAR score known		
Yes	464	75.8
No	148	24.2
APGAR score in 1st minute (*n* = 464)		
≤ 3	42	9.05
4–6	172	37.07
≥ 7	250	53.88
APGAR score in 5th minute (*n* = 464)		
4–6	103	22.20
≥ 7	361	77.80
Feeding Option		
Exclusive breastfeeding	568	92.81
Exclusive replacement feeding	4	0.65
Mixed feeding	40	6.54
Time of initiating breastfeeding (*n* = 608)		
≤ 1 h	296	48.68
> 1 h	261	42.93
Gestational Age in weeks (*n* = 568)		
< 37 wks	208	33.99
37wks- 42wks	350	57.19
> 42wks	10	1.63
Neonatal age at admission (in hours)		
< 24	424	69.3
24–168	105	17.2
168.1–672	83	13.6
Weight at admission in kg		
< 2.5 kg	237	38.7
≥ 2.5 kg	375	61.3

**Fig. 4:**
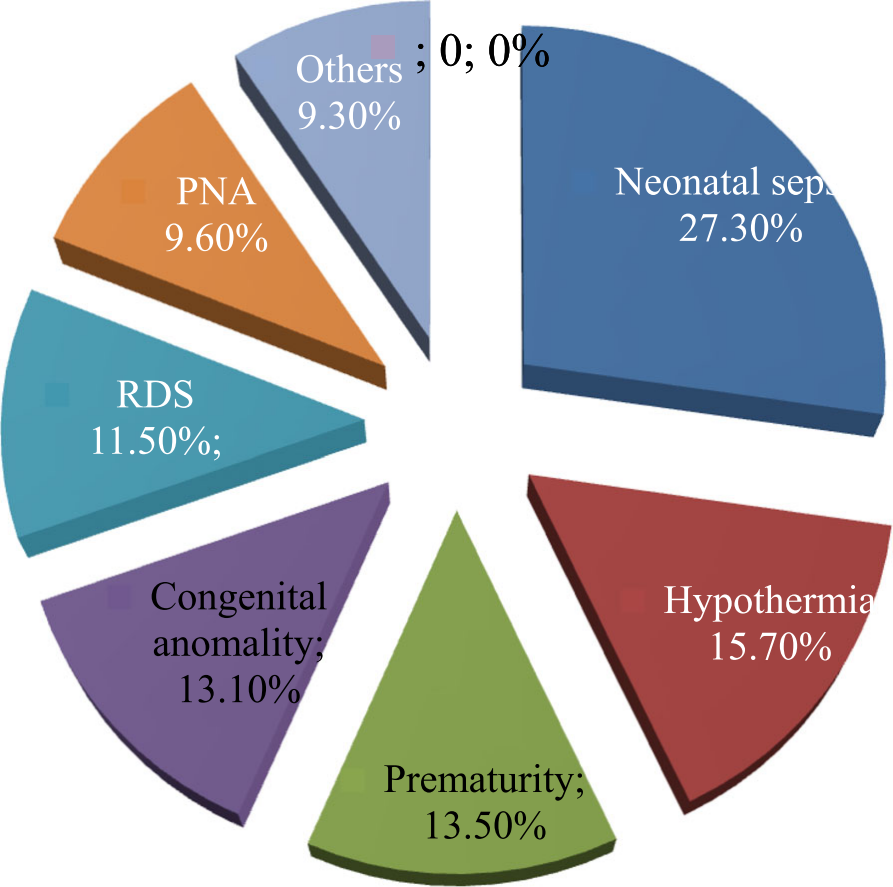
Reasons for neonatal admission at NICU in Amhara region referral hospitals, Northern Ethiopia, 2018

The top three reasons for neonatal admission at the NICU in the referral hospitals in Amhara national regional state were neonatal sepsis (27.3%), hypothermia (15.7%) and prematurity (13.5%) (Fig. 4).

### Neonatal mortality incidence rate and time to death

Admitted neonates had been followed up for a total of 4177.803 neonate-days. The proportion of neonatal death among admitted neonates was obtained to be 18.6% with 95% CI (15.7, 21.9) which is equivalent to the NMR of 186 per 1000 admitted neonates with 95% CI (157,219). Whereas, the neonatal mortality incidence rate (NMIR) was calculated to be 27 per 1000 admitted neonates with 95% CI (23, 33).

Among dead neonates, more than half (56.14%) of them have died within 24 h and about nine-tenth (90.35%) died in the early neonatal period. The overall cumulative probability of death was 28.89% with 95% CI (23.57, 35.12). The cumulative probabilities of death at the end of the first day and within the first 7 days were 10.85 and 20.30% respectively (Table 5).

**Table 5:** Failure and survival probability of neonates admitted at NICU in Amhara region referral Hospital, 2018 (*n* = 612)

Time in days	Total number at the beginning	Lost	Death	Death probability (%) with 95% CI	Survival probability (%) with 95% CI
0–1	612	44	64	10.85 (8.59,13.65)	89.15 (86.35,91.41)
2–7	504	272	39	20.30 (16.97,24.17)	79.70 (75.83,83.03)
8–28	193	182	11	28.89 (23.57,35.12)	71.11 (64.88,76.43)

As far as the influence of each primary diagnosis to neonatal death is concerned, low birth weight had attributed to 80 (70.18%) of neonatal deaths followed by preterm birth to 69 (60.53%), RDS to 47 (41.23%), neonatal sepsis to 28 (24.56%), PNA and PROM each to 15 (13.16%), hypoglycemia to 8 (7.02%), anemia to 7 (6.14%), MAS to 6 (5.26) and other minors such as congenital anomalies, jaundice, etc. to 7 (6.14%) neonatal deaths.

### Predictors of neonatal mortality

Initially, all variables had been entered to the bivariate Cox proportional hazard regression model. Based on the bivariate Cox proportional hazard regression analysis, maternal age, residence, mother’s educational status, number of ANC visit, TT vaccination, type of pregnancy, delivery complication, fetal presentation, maternal HIV status, parity, cry at birth, neonatal age at admission, neonatal sepsis, neonatal sex, and history of stillbirth had been associated with NM. Furthermore, variables with *P*-value of ≤0.2 in the bivariate analysis and with no missing values had been fitted to the final model. Ultimately, maternal age, maternal educational status, multiple pregnancies, and maternal HIV status had remained independent predictors of NM in the multivariable Cox proportional hazard regression analysis.

Accordingly, the risk of NM among neonates who born to mothers with advanced age was 2.60 times higher as compared to their counterparts (AHR = 2.60; 95% CI:1.44, 4.72). Likewise, the risk of mortality for neonates born to mothers who are unable to read and write was 1.40 times higher than that of neonates whose mothers are able to read and write (AHR =1.40; 95% CI:1.23, 2.44). Equally, multiple pregnancies increased the risk of NM by 3.96 folds as compared to the counterpart (AHR = 3.96; 95% CI: 2.10, 7.43). Similarly, neonates born to HIV positive mothers were 6.57 times more likely to experience death during the neonatal period than those neonates who born to sero-negative mothers (AHR =6.57: 95% CI: 2.53, 17.06) (Table 6).

**Table 6:** Predictors of neonatal death among neonates admitted in Amhara region referral hospitals, Northern Ethiopia, 2018 (*n* = 612)

Variables	Outcome of admission		Bivariate analysis	Multivariable analysis
	Death n (%)	Censored n (%)	CHR with 95% CI	AHR with 95% CI
Maternal age				
< 35 yrs.	72 (14.3)	432 (85.7)	1	**1**
≥ 35 yrs	42 (38.9)	66 (61.1)	3.82 (2.03,4.35)*	2.60 (1.44,4.72)*
Residence				
Urban	56 (14.6)	327 (85.4)	1	1
Rural	58 (25.3)	171 (74.7)	1.98 (1.27,2.66)*	1.22 (0.66,2.25)
Maternal education				
Able to read and write	55 (13.6)	350 (86.4)	1	**1**
Unable to read & write	59 (28.5)	148 (71.5)	2.54 (1.60,3.35)*	1.40 (1.23,2.44)*
Age at admission				
Early	111 (20.98)	418 (79.02)	7.08 (2.05,20.32)*	2.87 (0.84,9.87)
Late	3 (3.61)	80 (96.39)	1	1
Cry at birth				
No	80 (32.92)	163 (67.08)	4.84 (2.64,5.90)*	0.93 (0.49,1.78)
Yes	34 (9.21)	335 (90.79)	1	1
Number of ANC visit				
0	12 (31.58)	26 (68.42)	4.26 (1.79,7.04)*	0.78 (0.27,2.24)
1–3	76 (24.68)	232 (75.32)	3.02 (1.53,3.74)	0.91 (0.49,1.65)
≥ 4	26 (9.77)	240 (90.23)	1	1
Received TT vaccination				
Yes	82 (16.27)	422 (83.73)	1	
No	32 (29.63)	76 (70.36)	2.17 (1.33,3.01)*	2.14 (1.00,4.15)
Type of pregnancy				
Single-tone	82 (15.0)	466 (85.0)	1	**1**
Multiple	32 (50.0)	32 (50.0)	5.68 (2.95, 6.73)*	3.96 (2.10,7.46)*
Sex of neonate				
Male	58 (15.9)	307 (84.1)	1	1
Female	56 (22.7)	191 (77.3)	1.55 (0.96,2.01)	0.76 (0.48,1.19)
History of stillbirth				
No	84 (16.67)	420 (83.33)	1	
Yes	30 (27.78)	78 (72.22)	1.92 (0.94,2.18)	0.56 (0.31,1.02)
Delivery complication				
No	22 (7.6)	267 (92.4)	1	1
Yes	92 (28.5)	231 (71.5)	4.83 (2.47,6.30)*	1.30 (0.68, 2.48)
Neonatal sepsis				
No	86 (23.1)	286 (76.9)	1	1
Yes	103 (42.92	137 (57.08)	2.50 (1.81,5.27)*	0.69 (0.49,1.19)
Fetal presentation				
Cephalic	95 (17.0)	465 (83.0)	1	1
Non-Cephalic	19 (36.5)	33 (63.5)	2.82 (1.61,4.33)*	1.08 (0.58,2.02)
Mother HIV status				
Negative	106 (17.8)	491 (82.2)	1	**1**
Positive	8 (53.3)	7 (46.7)	5.29 (2.24,5.36)*	6.57 (2.53,17.06)*
Parity				
1	32 (12.2)	231 (87.8)	0.49 (0.36,0.86)*	1.12 (0.62,1.98)
2–4	59 (21.9)	211 (78.1)	1	1
≥ 5	23 (29.1)	56 (70.9)	1.47 (0.87, 2.28)	1.17 (0.56,2.44)

## Discussion

Neonatal mortality rate remains unacceptably high in most of resource limited countries including Ethiopia although under-five mortality has been declining substantially since 2000 [20]. Moreover, the proportion of neonatal deaths over under-five deaths is increasing time to time despite the governmental commitments. For example, in Ethiopia, the neonatal mortality rate was reported to be 29 per 1000 live births as of EDHS 2016 and was targeted to reduce to 10 per 1000 live births in 2019/20 in line with the target two of SDG 3 [9]. Paradoxically, the magnitude reported to be elevated (i.e. 30 per 1000 live births) according to mini EDHS 2019 [11]. Hence, it is among major current public health challenges which require appropriately designed strong studies to generate accurate and representative information so that stakeholders can apply evidence based interventions. Eventhough some studies had been conducted on neonatal mortality and predictors in Ethiopia, no large scale study including all referral hospitals of Amhara region was carried out, as to the investigators deep review. In addition, no prospective follow up studies had been done on neonatal death in the study area. Therefore, this study is aimed to address these gaps particularly to assess NMR, time to death and predictors among admitted neonates in referral hospitals in Amhara national regional state to provide basic information for any intervention aimed at improving neonatal health. .

The magnitude of NMR is higher than the national figure and the highest probability of neonatal death has been recorded in the early neonatal period. The risk of NM was higher among neonates whose mothers were HIV positive, uneducated, advance aged and gravid of multiple fetuses in the indexed pregnancy.

In the current study, NMR was 186 per 1000 admitted neonates. This finding is comparable with the study conducted in Wolaita Sodo referral hospital in southern Ethiopia (173) [21] and sub-urban hospital in Cameron (157) [22].

However, the current study findings demonstrated that the magnitude of NMR is higher than the result of other previous studies done in Iran- 91 [19], China- 12 [23], Suriname- 129 [24], Indonesia- 52 [25], Nigeria- 41 [7], Burkina Faso- 46.3 [26], Sudan- 30 [15], UNICEF 2018 report [1] and EDHS 2016 report- 29 [2]. It is also higher as compared to local studies conducted in various part of Ethiopia such as the Somali region of Ethiopia- 57 [27], Dilchora referral hospital in Dire Dawa- 114.4 [28], Jimma Zone- 35.5 [17], rural area of Eastern Ethiopia- 28.37 [29], Tigray, Ethiopia-62.5 [12] and Ethiopia-36.7 [10]. This discrepancy might be attributed to variation in study design. Unlike the current study, some previous studies were community-based [15, 17]. Hence, NM tends to be lower in the community-based studies where low-risk neonates were also included in the denominator, unlike the investigations conducted in the NICU. In the studies which have been undertaken at the NICU, like the current one, the denominators are all admitted neonates (i.e. the population at higher risk for death), and it is logical that the probability of neonatal death is likely to be higher among such population than the counterparts. Moreover, the disparity in magnitude could be accredited to variation in study settings. In other words, some previous studies were conducted in developed nations [24]. Empirical evidence supports that the overall health care system, as well as the survival rate of neonates, is much better in the developed countries than in resource-limited countries [1]. The implication is ascribed to the impact of the financial barriers for receiving basic obstetric and neonatal cares in economical poor countries including Ethiopia. In connection to this, one surprising point in the current study was that about 93.79% of respondents received at least one ANC. Similarly, more than 96% of participants gave birth at health facilities. This implies that the maternal health care service coverage was high and that was promising. However, about 52.78% of the mothers had experienced delivery complications. Moreover, nearly half (46.12%) of the neonates had poor 1st minute Apgar score. In addition, almost all reasons for neonatal admissions and neonatal deaths were found to be preventable. This questions the quality of health care services during the perinatal period. One most probable reason for such unacceptable poor quality of maternity care and poor neonatal health outcome is delays in accessing emergency obstetric and / or neonatal cares which include delays in decision to seek care (delays at the home), delays in reaching health facilities (delays at the journey) and delays in receiving appropriate care (delays at the health facilities). One known contributing factor for such delays is financial barrier which further seems true in the current study since about four-fifth (77.1%) of the respondents’ family had earned less than 139 US dollar monthly. The costs of essential maternal and child healthcare – whether formal or informal costs – are major barriers preventing the poorest and most excluded women and children getting the services they need. User fees have been shown to be highly regressive, with a particularly negative impact on the poor, reducing uptake of essential health services. Out-of-pocket (OOP) expenditure for healthcare during pregnancy and childbirth is still common in many low- and middle-income countries. Although the country, Ethiopia, endorsed the cost-free maternal and child health care services during pregnancy and child birth processes, most of the supplies and medications are unavailable in public health facilities at the time of request. Thus, women often have to pay formal or informal charges to receive care, and have to buy their own medicines and basic necessities, such as soap and clean sheets, when going into hospital to give birth. Thus, improving transportation, strengthening referral mechanisms, assuring ambulance distribution and maintenance policies, equipping health facilities with essential supplies, mobilizing resources for MNCH quality improvement, availing essential reproductive health commodities, facilitating inter-sectoral collaboration to address common maternal and newborn health problems and work for adequate budget allocation for MNCH would decrease the high magnitude of neonatal mortality. Furthermore, the discrepancy could be attributed to variation in the scope of the study. The former neonatal mortality reports were made based on the country’s aggregated findings [1, 2]. Thus, these averaged magnitudes are expected to be lower as compared to the death incidences from referral hospitals where near-missed-referred cases are more likely to be abundant. Also, the dissimilitude in mortality might be ascribed to variation in parent’s socio-demographic characteristics.

On the other hand, the magnitude of NMR in the current study is lower as compared to the findings of the studies done at University of Gondar referral hospital- 252 [30], Paul’s Hospital Millennium Medical College Addis Ababa- 232 [31], Pakistan Health Survey of 2014–680 [32] and University of Gondar comprehensive Specialized hospital (UoGCSH)- 288 [33]. The variation in the magnitude of NMR could be secondary to the difference in the scope of the study. It is observed that some other previous studies were conducted in a single centered institution which might have lacked representativeness unlike the current one [30, 31, 33]. The disparity also could be linked to discrepancy in sample size. In the former study conducted in Addis Ababa, the sample size was 216 which accounts only nearly one-third of the current one [31]. The difference in magnitude also might be related to respondent’s residency. In the previous study, about seven-tenths (70%) of the respondents were rural dwellers which is much greater than that of in the current study [32]. In this perspective, evidences exhibit that neonatal mortality tends to be higher among rural residents [34, 35]. This could be rationalized as rural residents usually could not get access to health-related information easily and health care services timely as equal as urban residents. Besides, the dissimilarity in NMR extent might be secondary to variation in the study population. Unlike the current study which recruited all admitted neonates disregard to their gestational age, some previous studies had entailed only preterm neonates [30, 33]. In connection to this, studies indicate that the risk of neonatal death is higher among preterm neonates than among term neonates [31, 36].

The current study exhibits that the overall cumulative survival probability of neonates at the end of the follow-up period was 71.11%. This result is lower as compared to the findings of a previous study done in Tigray, northern Ethiopia 93.96% [12]. This variation in survival probability of neonates might arise from the difference in the study population. In the former study, all live births (i.e. all alive neonates) had been recruited, whereas only admitted neonates (i.e. population at high risk) had involved in the current one. Thus, the survival probability is more likely to be higher among all live births than that of neonates admitted with some problems.

On the contrary, the survival probability of neonates in the current study is higher as compared to that of the study carried out at UoGCSH (57.14%) [33]. This dissimilarity might be accredited to variation in study population again. Accordingly, the study population in the previous study was premature neonates that pose a higher probability of death as compared to that of in the current study (i.e. all neonates irrespective of gestational age).

This study also shows that the cumulative survival probability of neonates at the end of the first day was 89.15%. This is lower than the previous study done at UoGCSH where the cumulative survival probability over 24 h was 96.71% [33]. As far as the result of these two studies compared, some sort of surprising findings are observed. In the first 24 h, the survival probability of neonates in the former study (96.71%) was higher than that of the current one (89.15%). Thereafter, the survival probability in the early neonatal period and overall cumulative period comes to be lower in the previous study (74.62 and 57.14% respectively) as compared to the current study (79.70 and 71.11% respectively). This implies that the probability of death among study population in a previous study (i.e. premature neonates) has highly increased as neonatal age advanced than that of the general admitted neonates.

In the current study, it was observed that the risk of death among neonates born to mothers with advanced age is more than two times higher as compared to their counterparts. This finding is also supported by other studies conducted in Sudan [15] and Suriname [24]. This could be related to the evidence-based facts that advanced maternal age is associated with certain pregnancy-related risks including poor neonatal outcome [37]. Therefore, efforts need to be collaborated to minimize the risky conditions such as pregnancy at advanced maternal age.

In many circumstances, maternal educational status matters obstetric and perinatal outcomes [10]. Studies indicated that the outcome of pregnancy among educated women is more likely to be better than among uneducated ones [38]. The current study had come up with findings which support this fact. Accordingly, the risk of death for neonates born to mothers who are unable to read and write was 1.40 times higher as compared to those neonates whose mothers can read and write. This aligns with the findings of previous studies done in different settings in Ethiopia [10, 34]. This could be explained by the role of education on the maternal level of apprehensive. Accordingly, educated mothers have a better awareness of the healthcare-related information and thus, might behave and act as per the standard of health care service utilization in a symphonious manner with their caregiver. Moreover, educated mothers are more likely to search health-related information in a variety of other ways such as by attending mass media, browsing internets and reading magazines. Consequently, they are likely to evolve with accurate health-related information enough to make appropriate decisions and to take corrective actions even in the emergency. Surprisingly, nearly two-fifth (38.72%) of the respondents in the current study had never attended any formal education at the time of interview. Meanwhile, being unable to read and write exhibited to be the independent predictor of neonatal mortality. The finding implies that all stakeholders need to work more on the gender inclusive education policy which promotes all females to be engaged in formal education. Similarly, about 40.81% of fathers of neonates had never been educated through formal education. Hence, the finding also entails the need of information, education, and communication to improve the community awareness of neonatal death risk factors and danger signs.

Also, neonates born to HIV positive mothers were 6.57 times more likely to die during the neonatal period as compared to those born to sero-negative mothers. This is following a systematic review study [8]. This is also in line with a study conducted at University of Gondar Referral Hospital, Ethiopia [30]. This might be related to the effect of HIV infection on the maternal overall health condition. And it is evidence-based fact that the fetal health condition is directly or indirectly influenced by maternal health status [39]. In other words, the maternal and fetal general condition is strongly interlinked to each other. Thus, if the maternal immunity and other conditions are compromised by HIV infection, the fetal health condition would be endangered too, which the jeopardized fetal health condition would more likely lead to neonatal death. The finding, herein, suggests that offering integrated PMTCT services to MNCH programs would decrease neonatal mortality rate. The result also further alerts the health care providers to be vigilant enough to screen, diagnose and treat maternal infections. According to the current study finding, the risk of neonatal mortality is more than three times higher in multiple pregnancies than single tones. This finding is supported by previous studies done in different settings [17, 19, 26, 30, 40]. This could be attributed to the effect of multiple pregnancies in life-threatening perinatal outcomes. Multiple pregnancies usually carry a higher risk of prematurity, premature rupture of membrane, abnormal presentation and amniotic fluid abnormality and IUGR which all may increase the probability of neonatal death [41–43]. Hence, as multiple gestations is one of the high risk pregnancies and is an independent predictor of neonatal death, it demands close monitoring and care.

## Limitations

This study recruited all admitted neonates who were at higher risk of death. Thus, the NMR reported from this study could be overestimated as compared to other reports made by taking all live births as a denominator. In this study, the measurement of some variables might have been committed to recall bias. Of course, we had executed certain efforts to minimize such potential recall biases. First, the questionnaire had been well devised in such a way that it was structured from simple to complex and recent to past in chronological order. It was also translated to local language. In addition, we had evaluated the tool by conducting a pretest prior to the actual data collection. Second, we had delivered trainings for the data collectors and supervisors on the strategies of minimizing recall bias. Thus, the interviewers had been told to assure that each question is clearly understood by the respondents before the responses, to inform the local events that may trigger the respondents’ recalling capacity, to give participants sufficient time to recall long term memory, to verify with any record or any other potential source of information such as family members, intimate friends and any other persons who were around at the time of event, and to use multiple source of information like crosschecking the documented records and contacting the concerned health care providers for uncertainties in documentations.

## Conclusion

The neonatal mortality incidence rate in the study setting was higher than the national figure, and the highest probability of neonatal deaths has been recorded in the early neonatal period. Advanced maternal age, uneducated maternal status, positive maternal HIV status, and multiple pregnancies were the independent predictors for neonatal mortality. Thus, stakeholders need to consider the distinguished risky time and the identified independent risk factors of neonatal death while they design strategies to reduce the high mortality rate.

## Data Availability

While ethics statement has been stated, we have agreed and signed in order not to publish the raw data retrieved from the information of the mothers. However, the data sets collected and analyzed for the current study are available from the corresponding author and can be obtained on a reasonable request.

## Abbreviations

AHR: Adjusted Hazard Ratio
ANC: Ante-Natal Care
APGAR: Appearance, Pulse, Grimes Activity and Respiratory
C/S: Cesarean Section
CHR: Crude Hazard Ratio
CI: Confidence Interval
EDHS: Ethiopia Demographic Health Survey
HIV: Human Immunodeficiency Virus
HR: Hazard Ratio
IDM: Infants of Diabetes Miletus
IQR: Inter Quartile Range
MAS: Meconium Aspiration Syndrome
MOH: Minister of Health
NICU: Neonatal Intensive Care Unit
NM: Neonatal Mortality
NMIR: Neonatal Mortality Incidence Rate
NMR: Neonatal Mortality Rate
PNA: Perinatal Asphyxia
PROM: Premature Rupture of Membrane
RDS: Respiratory Distress Syndrome
SD: Standard Deviation
SDG: Sustainable Development Goal
SGA: Small For Gestational Age
TT: Titanus Toxoid
UNICEF: United Nations International Children’s Emergency Fund
UoGCSH: University of Gondar Comprehensive Specialized Hospital
